# The complete chloroplast genome sequence of *Kadsura interior*

**DOI:** 10.1080/23802359.2019.1710297

**Published:** 2020-01-14

**Authors:** Yupin Fu, Yunqing Li, Wei Chen, Yi Wang

**Affiliations:** Laboratory of Forest Plant Cultivation and Utilization, Yunnan Academy of Forestry, Kunming, Yunnan, People’s Republic of China

**Keywords:** *Kadsura interior*, chloroplast, Illumina sequencing, phylogenetic analysis

## Abstract

The first complete chloroplast genome (cpDNA) sequence of *Kadsura interior* was determined from Illumina HiSeq pair-end sequencing data in this study. The cpDNA is 153,201 bp in length, contains a large single-copy region (LSC) of 85,774 bp and a small single-copy region (SSC) of 18,077 bp, which were separated by a pair of inverted repeats (IR) regions of 24,673 bp each. The genome contains 129 genes, including 85 protein-coding genes, 8 ribosomal RNA genes, and 37 transfer RNA genes. The overall GC content of the whole genome is 39.6%, . The further phylogenomic analysis showed that *K. interior* and *Kadsura coccinea* clustered in a clade in Schisandraceae family.

*Kadsura interior* is the species of the genus *Kadsura* within the family Schisasndraceae. It is mainly distributed in the southwest Yunnan of China (Chen et al. 1996). Their roots and stems have been used as a traditional medicine to promote blood circulation, activate collaterals, and relax muscles (Chen et al. 2002). It also is generally used in the treatment of menstruation, hemopenia yellow, numbness, and paralysis (Zhou et al. 2008). The lignans isolated from *K. interior* have anti-tumor activity and anti-HIV virus activity (Chen et al. 1997). Therefore, *K. interior* has huge potential medicinal value. However, there have been no genomic studies on *K. interior*.

Herein, we reported and characterized the complete *K. interior* plastid genome. The GenBank accession number is MN698966. One *K. interior* individual (specimen number: 201905063) was collected from Kunming arboretum, Yunnan Academy of Forestry, Yunnan Province of China (25°14′23′′ N, 102°75′18′′ E). The specimen is stored at Yunnan Academy of Forestry Herbarium, Kunming, China and the accession number is YAFH0012983. DNA was extracted from its fresh leaves using DNA Plantzol Reagent (Invitrogen, Carlsbad, CA).

Paired-end reads were sequenced by using the Illumina HiSeq system (Illumina, San Diego, CA). In total, about 25.2 million high-quality clean reads were generated with adaptors trimmed. Aligning, assembly, and annotation were conducted by CLC de novo assembler (CLC Bio, Aarhus, Denmark), BLAST, GeSeq (Tillich et al. 2017), and GENEIOUS v.11.0.5 (Biomatters Ltd, Auckland, New Zealand). To confirm the phylogenetic position of *K. interior*, the other four species of Schisandraceae family from NCBI were aligned using MAFFT v.7 (Katoh and Standley 2013). The Auto algorithm in the MAFFT alignment software was used to align the seven complete genome sequences and the G-INS-i algorithm was used to align the partial complex sequences. The maximum-likelihood (ML) bootstrap analysis was conducted using RAxML (Stamatakis 2006); bootstrap probability values were calculated from 1000 replicates. *Nuphar advena* (DQ354691) and *Nuphar longifolia* (MH050795) served as the out-group.

The complete *K. interior* plastid genome is a circular DNA molecule with the length of 153,201 bp, contains a large single-copy region (LSC) of 85,774 bp and a small single-copy region (SSC) of 18,077 bp, which were separated by a pair of inverted repeat (IR) regions of 24,673 bp each. The overall GC content of the whole genome is 39.6%, and the corresponding values of the LSC, SSC, and IR regions are 38.8, 35.0, and 42.9%, respectively. The plastid genome contained 129 genes, including 85 protein-coding genes, 8 ribosomal RNA genes, and 37 transfer RNA genes. Phylogenetic analysis showed that *K. interior* and *Kadsura coccinea* clustered in a unique clade in the Schisandraceae family (Figure 1). The determination of the complete plastid genome sequences provided new molecular data to illuminate the Schisandraceae family evolution.

**Figure 1.**
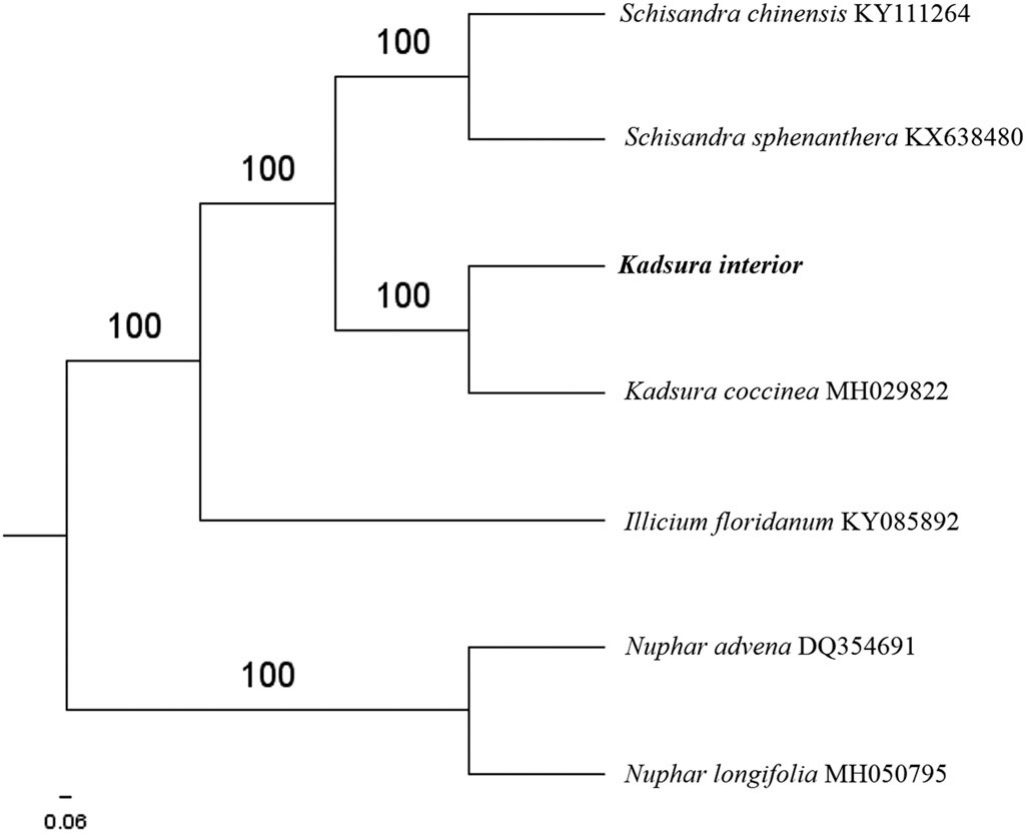
The maximum-likelihood tree based on the five chloroplast genomes of *Schisandraceae* family. The bootstrap value based on 1000 replicates is shown on each node.
